# The novel diagnostic markers for systemic lupus erythematosus and periodontal disease

**DOI:** 10.3389/fimmu.2025.1614044

**Published:** 2025-07-22

**Authors:** Xuedi Cheng, Jinfeng Zhang, Jiali Hou, Xiaocui Han, Bin Han, Jun Zhou, Zhongjun Wang, Junzheng Wang

**Affiliations:** ^1^ Department of Clinical Laboratory, The Affiliated Hospital of Qingdao University, Qingdao, Shandong, China; ^2^ Department of Clinical Laboratory, Qingdao Women and Children’s Hospital, Qingdao, Shandong, China; ^3^ Department of Oral and Maxillofacial Surgery, Qingdao Haici Medical Group, Qingdao, Shandong, China; ^4^ Department of Pathology, The Affiliated Hospital of Qingdao University, Qingdao, Shandong, China

**Keywords:** systemic lupus erythematosus (SLE), periodontal disease (PD), targeted drug, hub genes, single-cell sequencing, molecular docking

## Abstract

**Background and aims:**

Systemic lupus erythematosus (SLE) is one of the most prevalent systemic autoimmune diseases, characterized by aberrant activation of the immune system that leads to diverse clinical symptoms; periodontal disease (PD) is an inflammatory oral disorder caused by immune-mediated damage against subgingival microflora. Although clinical evidence suggests a potential association between SLE and PD, their shared pathogenic mechanisms remain unclear. This study aims to explore common genetic markers in SLE and PD that hold diagnostic and therapeutic implications.

**Methods:**

Microarray datasets for systemic lupus erythematosus (SLE) and periodontal disease (PD) were obtained from the Gene Expression Omnibus (GEO) database. Module genes between the two diseases were screened using Weighted Gene Co-expression Network Analysis (WGCNA), and module genes overlapping between the significant correlation modules of GSE61635 and GSE16134 were identified. Functional enrichment analyses of genes within overlapping modules and their significantly correlated associated modules were performed using Gene Ontology (GO) and Kyoto Encyclopedia of Genes and Genomes (KEGG) pathway analysis. Overlapping module genes underwent differential expression analysis in GSE16134. A diagnostic model was constructed using the Random Forest (RF) machine learning technique under Receiver Operating Characteristic (ROC) curve assessment, which top 10 key genes were screened and analyzed for differential expression across three datasets (GSE61635, GSE10334, and GSE50772) to identify hub genes. Protein-protein interaction (PPI) network analysis was conducted to explore relationships between hub genes. CIBERSORT and Gene Set Variation Analysis (GSVA) were used to evaluate the correlation between shared hub genes and immune infiltration patterns as well as metabolic pathways. Finally, hub genes were validated using additional datasets, single-cell RNA sequencing (scRNA-seq) data, and immunohistochemistry (IHC) experiments.

**Results:**

Using WGCNA, we identified significant correlation modules and overlapping module genes, which were subjected to differential expression analysis in different datasets. Further, 4 hub genes were screened and successfully used to build a prognostic model. Those shared hub genes were associated with immunological and metabolic processes in peripheral blood. The additional datasets, scRNA-seq and IHC results verified that LY96 and TMEM140, possessing the promising diagnostic and therapeutic performance.

**Conclusion:**

LY96 andTMEM140 can be used as new diagnostic and therapeutic markers for SLE and PD.

## Introduction

Systemic lupus erythematosus (SLE) is a systemic autoimmune disease that is characterized by a breakdown in immune tolerance and an exaggerated autoimmune response. There has been a rise in the prevalence of atypical, early, or comorbid cases of SLE (1). The characteristic clinical manifestations of SLE encompass the presence of erythematous macules on the skin and the involvement of multiple organs, predominantly observed in young females (2, 3). The etiology of SLE is a complex phenomenon that has yet to be fully elucidated. It is believed to involve a combination of genetic predisposition, environmental exposure, gender, and endogenous triggers (4).

Periodontitis is a chronic inflammatory noncommunicable disease that impacts the entire periodontium and leads to irreversible damage. The immune and inflammatory response to the presence of bacteria, specifically gram-negative bacteria, in the gingival sulcus results in the periodontal attachment loss (5). A multitude of factors, including genetic, metabolic, immunological, and inflammatory factors, are associated with the progression of periodontitis. Thus, it is imperative to establish dependable, unbiased, and replicable biomarkers for the timely detection of periodontitis (6).

A substantial body of research has demonstrated a potential correlation between periodontal diseases and autoimmune conditions, including rheumatoid arthritis and SLE, such as IL-1and IL-18, in the pathogenesis of both conditions, contributing to tissue destruction, SLE exhibits an enhanced ability to reduce the periodontal αKG levels, thereby promoting the inflammation and bone loss of periodontal disease (PD), and Periodontitis might induce the overactivation of the immune response in SLE by maintaining high expression of TLR, which would then lead to the accelerated occurrence and progression of the autoimmune response (7–10). Despite the increasing body of evidence indicating a close association between SLE and PD, previous studies primarily adopted a clinical perspective and failed to elucidate the underlying molecular mechanisms at the genetic level. Additionally, there is a lack of research on targeted drugs and their mechanisms for patients with comorbidity. Therefore, it is necessary to conduct more studies that focus on common molecular mechanisms in order to enhance our understanding in this area.

The objective of this study was to employ unbiased bioinformatics methodologies to systematically elucidate the molecular signatures associated with the pathogenesis of systemic lupus erythematosus (SLE) and periodontal disease (PD). This investigation specifically aimed to identify key dysregulated genes, immune cell profiles, and functional pathways and focuses on three mechanistic dimensions—immune-metabolic crosstalk, inflammatory signaling integration, and transcriptional dysregulation—to reveal shared pathological bases of SLE and PD via multi-omics integration, which including differential gene expression (DEG) profiling, immune infiltration assessment, gene set variation analysis (GSVA), and molecular-ligand docking—we endeavored to reveal both shared and distinct biological mechanisms underlying these two immune-related disorders. The findings were subsequently validated through additional datasets, single-cell RNA sequencing and immunohistochemistry (IHC) conducted on clinical specimens from SLE patients and healthy controls. Ultimately, the primary aim was to identify and validate novel biomarker candidates that could potentially serve as targets for enhancing the diagnosis and therapeutic approaches for SLE and PD.

## Materials and methods

### Data selection

The search terms “lupus” or “SLE” and “Periodontal disease” or “periodontitis” were utilized to retrieve gene expression profiles from the GEO (http://www.ncbi.nlm.nih.gov/geo) database (11). The search was filtered to include only samples obtained from peripheral blood. The obtained dataset is filtered based on the following criteria: Firstly, the gene expression profiling must include both cases and controls. Second, the organization used for sequencing should be peripheral blood mononuclear cells (PBMC) for SLE and PD. Thirdly, it is crucial to ensure the accuracy of the Weighted Gene Co-expression Network Analysis (WGCNA) by having a minimum sample size of 15 in each group. Fourth, these datasets must provide the processed data or raw data that could be used for reanalyzation. Finally, the GEO datasets GSE50772, GSE61635, GSE16134, GSE10334, GSE135779 and GSE174609 were chosen (**Table 1**). The Series Matrix Files provided by the contributors include the normalized data processed by MAS5 algorithm. We subsequently conducted log2 transformation on the gene expression profiling data and associated the probes with their respective gene symbols based on the annotation document of the corresponding platforms. Lastly, the gene matrix with row names as sample names and column names as gene symbols were obtained for subsequent analyses.

**Table 1:** Summary of those six GEO datasets involving SLE and PD patients.

ID	GSE number	Platform	Samples	Source types	Disease	Group
1	GSE61635	GPL570	99 patients and 30 controls	PBMC	SLE	Training cohort
2	GSE16134	GPL570	241patients and 69 controls	PBMC	PD	Training cohort
3	GSE50772	GPL570	61 patients and 20 controls	PBMC	SLE	Validation cohort
4	GSE10334	GPL570	183patients and 64 controls	PBMC	PD	Validation cohort
5	GSE135779	GPL20301	40 patients and 16 controls	PBMC	SLE	Validation cohort
6	GSE174609	GPL20795	8 patients and 4 controls	PBMC	PD	Validation cohort

### Construction of weighted gene co−expression network analysis

WGCNA was conducted on the GSE61635 and GSE16134 datasets to identify gene modules. The “pickSoftThreshold” function from the WGCNA package (http://www.genetics.ucla.edu/labs/horvath/CoexpressionNetwork/Rpackages/WGCNA) was utilized for this purpose. The genes ranking in the top 5000 of the median absolute deviation in the corresponding expression matrix were selected for WGCNA. After removing missing values and genes with zero variance, the remaining values were selected to construct an adjacency matrix using the scale-free topology criterion, the appropriate soft power parameter, denoted as b, was determined utilizing the “pickSoftThreshold” function within the WGCNA package to assess the range of threshold values (power = 1 - 20), adhering to the criteria for establishing a scale-free network, these criteria are designed to achieve a balance between the scale-free characteristics of the network and its biological relevance, thereby ensuring that the network accurately mirrors the attributes of the biological system while preserving an adequate level of gene interactions. It is important to note that setting the threshold too high may yield an excessively sparse network, while a threshold that is too low could lead to the incorporation of spurious connections, thus, the soft threshold b was set to 0.9 for the WGCNA analysis (scale-free R2 = 0.9). Subsequently, the selected soft power value b, along with the gene correlation matrix derived from Pearson correlation analysis for all gene pairs, was employed to construct the adjacency matrix. This matrix was computed using formula. Following this, the topological overlap matrix (TOM) and its corresponding dissimilarity (1 - TOM) were derived from the adjacency matrix. A hierarchical clustering dendrogram was then generated, allowing for the categorization of genes with similar expression patterns into distinct modules. Utilize the cutreeDynamic function to perform dynamic tree cutting, configuring the parameters with minClusterSize set to 100 and deepSplit set to 2. Subsequently, compute the module eigengenes using the module Eigengenes function, which encapsulate the overall expression patterns of the identified modules. Following this, assess the correlation (moduleTraitCor) and significance (moduleTraitPvalue) between the module eigengenes and the phenotype variable (Treat). Identify the modules that exhibit high absolute correlation values alongside statistically significant p-values (generally p < 0.05) as the primary modules of interest. The expression profiles of each module were subsequently summarized using the module eigengene (ME), and the correlation between the ME and clinical features was assessed. Consequently, modules exhibiting a high correlation coefficient with clinical features were prioritized, and the genes within these modules were selected for further analysis. In this investigation, to identify key modules, the minimum module size was determined at 30, and the cut height was set at 0.25.

### GO and KEGG functional enrichment analysis

Utilizing the R software package, Gene Ontology (GO) biological process (BP), cellular component (CC), molecular function (MF), and Kyoto Encyclopedia of Genes and Genomes (KEGG) pathway enrichment analyses were performed on the positive module genes identified from the Weighted Gene Co-expression Network Analysis (WGCNA) of systemic lupus erythematosus (SLE) and periodontal disease (PD). Additionally, following the execution of GO/KEGG of Gene analyses on overlapping module genes, the primary functions of these gene sets were elucidated through GO and KEGG pathway enrichment analyses.

### Hub genes screening and validation based on the machine learning algorithm

The SLE data set GSE61635 and PD data set GSE16334 were chosen as the training sets. We utilized an additional SLE dataset, GSE50772, and a PD dataset, GSE10334, for the purpose of gene expression level validation, and an additional SLE dataset, GSE135779, and a PD dataset, GSE174609, for the purpose of single cell level validation. The R packages RF, XGB, SVM, and GLM were employed to identify and validate hub genes (12–14), All algorithms were executed utilizing the train() function from the caret package, with parameter optimization conducted via five-fold repeated cross-validation (repeatedcv).To better comprehend the machine learning models, the R package “DALEX” is used to illustrate the residual distributions and feature significance among the four machine learning models. The prognostic efficiency of the algorithm was assessed using receiver operating characteristic (ROC) curves, and the AUC value demonstrates that the RF algorithm outperformed the other algorithms.

### PPI network construction

A protein-protein interaction (PPI) network was established utilizing GENEMANIA (http://genemania.org/search/) for hub genes to evaluate the functions of these genes.

### Correlation analysis was conducted to examine the relationship between hub gene expression and immune infiltration

CIBERSORT, a widely utilized deconvolution algorithm, is employed to annotate the genomes of various immune cell types within the microenvironment (15, 16). The objective of using the CIBERSORT algorithm is to analyze the proportion of 22 immune cells in the peripheral blood samples obtained from the GSE61635 and GSE16134 datasets. A CIBERSORT p-value < 0.05 was considered statistically significant and included in the analysis. The Pearson correlation coefficient was used to ascertain the correlation between hub genes and immune-infiltrated cells. The visualization of the data was performed using the Boxplot and pheatmap R packages.

### Correlation analysis was conducted to examine the expression of overlapping module genes in relation to metabolic pathways

Gene Set Variation Analysis (GSVA) is a non-parametric and unsupervised method for estimating the changes in specific gene sets (17, 18). The activities of the KEGG hallmark pathways were quantified with the GSVA R package to find the related metabolic pathways in SLE and PD. In this part, p < 0.05 was regarded as statistically significant, and take veen intersection for the difference pathway. Pearson correlation coefficient was used to determine the correlation between overlapping module genes and metabolic pathways. The Pheatmap R package was utilized for data visualization.

### Isolation and library preparation of human peripheral blood mononuclear cells

#### GSE174609

Peripheral blood samples derived from Homo sapiens were collected in plastic blood collection tubes containing EDTA. PBMCs for scRNA-seq were isolated using SepMate (Stemcell Technologies Inc.) within 30 min of collection according to the manufacturer’s instructions. Briefly, density gradient medium and diluted blood samples were added to a SepMate tube. After carefully mixing the medium and samples, the tubes were centrifuged at 1200 × *g* for 10 min. The top layers were poured into a new tube and washed twice with phosphate-buffered saline containing 2% fetal bovine serum. The tubes were then centrifuged at 300 × *g* for 8 min at room temperature. Libraries were prepared using the chromium controller according to the 10× chromium Next GEM Single Cell 3′ v3.1 protocol. The cell suspension was mixed with the master mix and loaded with Single Cell 3′ v3.1 Gel Beads and Partitioning Oil into a chromium Next GEM chip G. RNA transcripts from single cells were uniquely barcoded and reverse-transcribed within droplets. cDNA molecules were pooled and then subjected to end repair, addition of a single ‘A’ base, and ligation of the adapters. Next, the products were purified and enriched using PCR to create a final cDNA library. Finally, the libraries were sequenced using the Illumina HiSeq platform according to the read length provided in the user guide.

#### GSE135779

Peripheral blood samples derived from Homo sapiens were collected in plastic blood collection tubes containing EDTA. The PBMCs were obtained after centrifugation and washing the density gradient medium and diluted blood samples. Viability was determined using trypan blue staining and measured on a Countess FLII. Flow Cytometry Cells were stained with fluorochrome-labeled antibodies to the following surface markers: CD3 (UCHT1, 1:100, BD Biosciences), CD8a (RPA-T8, 1:100, BioLegend), and CD14 (MSE2; 1:100, BD Biosciences). Subsequent to surface staining and staining with live/dead fixable dye (Aqua, 1:1000, Thermo-Fisher), cells were fixed and permeabilized according to the manufacturer’s instructions (Cytofix/Cytoperm and Perm/Wash Buffer; BD Biosciences), and stained for 30 min on ice for Granzyme A (GB9, 1:50, BioLegend), Granzyme B (GB11, 1:50, BioLegend), Perforin (B-D48, 1:50, BD Biosciences), and ISG15 (IC8044P, 1:50, R&D Systems). The stained cells were acquired with LSR Fortessa X-20 (BD) and analyzed with FlowJo software (BD). Briefly, 12,000 cells were loaded for capture onto the Chromium System using the v2 single cell reagent kit (10X Genomics). Following capture and lysis, cDNA was synthesized and amplified (12 cycles) as per manufacturer’s protocol (10X Genomics). The amplified cDNA was used to construct an Illumina sequencing library and sequenced on a single lane of a HiSeq 4000.

### Dataset download

Samples were downloaded from the GEO (https://www.ncbi.nlm.nih.gov/geo/) database using the GEO query. The samples in the expression profiling dataset GSE135779 and GSE174609 dataset are all derived from Homo sapiens. GSE135779 dataset contains 56 blood samples, including 16 whole blood samples from healthy donors and 40 whole blood samples from SLE patients. GSE174609 dataset contains 12 blood samples, including 4 whole blood samples from healthy donors and 8 whole blood samples from PD patients. Datasets are standardized with annotated probes and other data cleaning operations, the data processing was performed using the Seurat package (version 4.3.0) in R Studio (19).

### Analysis of scRNA-seq data

Single-cell sequencing technology is a robust method for profiling individual cells and gaining insights into cellular-level biological processes (20, 21). Single cell sequencing data quality control was performed as a necessary step. A cell-by-gene count matrix was constructed for analysis. To enhance data quality, cells exhibiting fewer than 500 unique molecular identifiers (UMIs) or exceeding 20,000 UMIs, as well as those with more than 20% of their gene expression attributed to mitochondrial genes, were excluded from the dataset. Furthermore, cells that expressed fewer than 250 genes or more than 5,000 genes were also removed. Additionally, cells demonstrating less than 80% complexity, defined as the ratio of the number of genes detected per UMI after log transformation, were filtered out, as these may represent specific cell types, artifacts, or contaminants. Based on the variance stabilization transformation (VST), the analysis focused on the first 2000 highly variable genes from each sample after normalization. The initial 2000 genes with high variability were subjected to scaling using the ScaleData function, and the dimensionality of the principal component analysis (PCA) was reduced using the RunPCA function. We selected a dimension of 50 and employed the FindNeighbors and FindClusters functions to cluster the cells into 22 distinct cell populations. Then the function of RunUMAP was performed for the visualization. For the annotation of cell populations, we utilized signatures of T, B, NK, m-DC, p-DC, Mono, and PCs cell annotation. Additionally, the VlnPlot function was employed to validate the spatial distribution and expression patterns of potential biomarkers across various cell types.

The Seurat R package (version 4.3.0) was used for data integration, scaling, clustering, and visualization. The remaining count data was normalized using the SCTransform function. To integrate with the largest dataset in the samples, the FindIntegration Anchors and Integrate Data functions were applied, with this dataset serving as the reference dataset. Subsequently, scaling and principal component analysis (PCA) were performed using the ScaleData and RunPCA functions, respectively. The first 30 components were used to construct UMAP dimensionality reduction and shared nearest neighbor (SNN) graphs through the RunUMAP and FindNeighbors functions. Community detection was performed using a graph-based modular optimization algorithm based on the Louvain method to identify cell clusters, with a resolution set to 0.6, effectively distinguishing cell types and detecting subtle molecular signals. Cell identity markers were determined using the “FindAllMarkers” function, where genes with a log fold change threshold greater than 0.5 and a minimum percentage greater than 0.25 were classified as significant differentially expressed genes (DEGs). Cell types were annotated based on an established set of marker genes for human immune cells.

### IHC analysis

#### Patient and sample collection

All tissue samples for immunohistochemistry (IHC) staining were collected from 40 patients with SLE (20 lupus nephritis and 20 lupus skin diseases) and 40 control individuals (20 chronic nephritis and 20 general dermatitis) between January 2013 and December 2023 at the Affiliated Hospital of Qingdao University. We received the written informed consent from participants in this study. The clinical diagnosis of patients with lupus nephritis (LN) was confirmed through renal biopsy, also with a specific index called SLEDAI. All patients included in the study met the 1997 American College of Rheumatology revised classification criteria for systemic lupus erythematosus (SLE) and were confirmed to have lupus nephritis (LN) through pathological examination using light microscopy, immunofluorescence, and transmission electron microscopy. Our research complied with the ethical principles outlined in the Declaration of Helsinki, Patient informed consents were obtained and approval of the internal review and ethics boards of the Affiliated Hospital of Qingdao University was also acquired.

#### Tissue preparation and immunohistochemical staining

The specimens underwent fixation in 10% neutral buffered formalin for a duration of 24 hours, followed by dehydration using a series of graded ethanol solutions. Subsequently, they were stained with xylene and embedded in paraffin. The resulting sections were prepared at a thickness of 4μm. These sections were then affixed to poly-L-lysine coated glass slides and subjected to a baking process at 60°C for a period of 2 hours.

The tissue sections were subjected to de-waxing using xylene and subsequently rehydrated through a gradient of ethanol and water. The antigen retrieval solution, consisting of a citrate buffer at pH 6.0, was heated for a duration of 15 minutes and allowed to cool to room temperature for 20 minutes. Following this, the sections were incubated with a 3% hydrogen peroxide solution at room temperature for 10 minutes, after which they were rinsed three times with phosphate-buffered saline (PBS), with each rinse lasting 5 minutes.

We used H2O2 to block endogenous peroxides and conducted antigenic thermal repair. The slices were blocked with fetal bovine serum (FBS) at 37°C for 30 minutes, then, the sections were probed with primary antibodies against TMEM140 (rabbit, 1:100, CSB-PA023710LA01HU, CUSABIO) and LY96 (rabbit, 1:500, 822065, zeobio) overnight at 4 ^°^C. Next, the sections were re-probed with secondary antibodies to goat anti-rabbit IgG (1:5000, CSB-PA992375, CUSABIO) for 1 h at room temperature. Finally, DAB H2O2 was applied to the slices to color the sites of the antibody binding for approximately 10 minutes. Subsequently, the slices were counterstained with hematoxylin, followed by the conventional slide-sealing process. A minimum of five distinct images, which do not overlap within the field of view, were acquired using an optical microscope (Olympus, Japan) at a magnification of 400×, all parameters related to image acquisition, including exposure time and gain, are maintained consistently to ensure comparability across groups. The analysis was performed utilizing the Image J software. Initially, the image was transformed into an 8-bit grayscale format, followed by the application of a uniform threshold to eliminate the background. Subsequently, the “Color Segmentation” tool was employed to differentiate between the DAB positive signal, represented in brown, and the hematoxylin counterstain, depicted in blue. The grade of staining intensity and immunohistochemical staining results (IRS) were analyzed by three pathologists with intermediate professional titles. The staining intensity is divided into different grades according to the depth of color of cytoplasmic staining in sections (Dyeing intensity: 0 (negative), 1+ (weak), 2+ (medium), 3+ (strong)).

### Small molecule agent screening and analysis of molecular-ligand docking

The CTD database (https://ctdbase.org) is a database that integrates data on interactions between a large number of chemicals, genes, functional phenotypes, and diseases, facilitating the study of disease-related environmental exposures and potential mechanisms of drug action (22). Its primary purpose is to investigate the functional associations between genes, small molecule compounds and diseases. The 3D structure of the drug core component was obtained from the PubChem database (https://pubchem.ncbi.nlm.nih.gov) (23). The primary protein structures of the target genes were acquired from The Protein Data Bank (PDB) (http://www.rcsb.org). AutoDock Tools software (version 4.2) was employed for the molecular docking of the primary targets with small molecule compounds (24, 25). The visualization of the binding activities between small molecule compounds and targets was conducted by analyzing the docking models using Pymol software (http://www.pymol.org) (26).

## Results

### Weighted gene co-expression network analysis of SLE and PD

**Figure 1:**
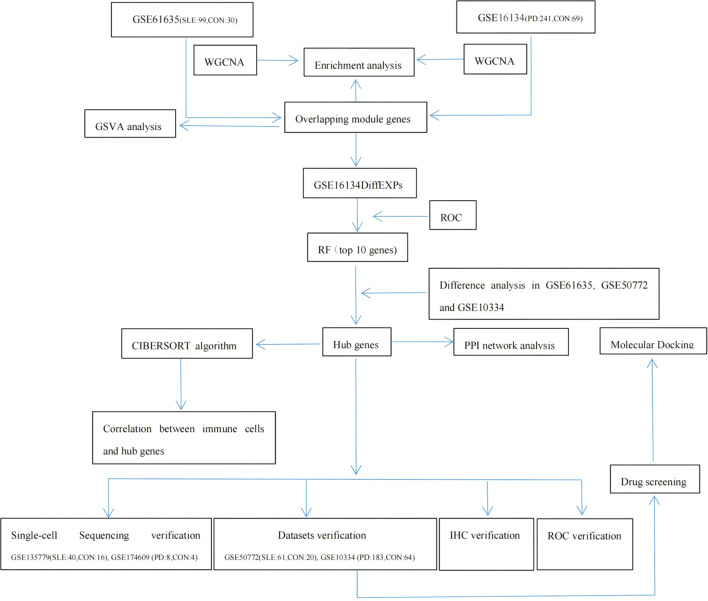
Flowchart of Investigation.

**Figure 2:**
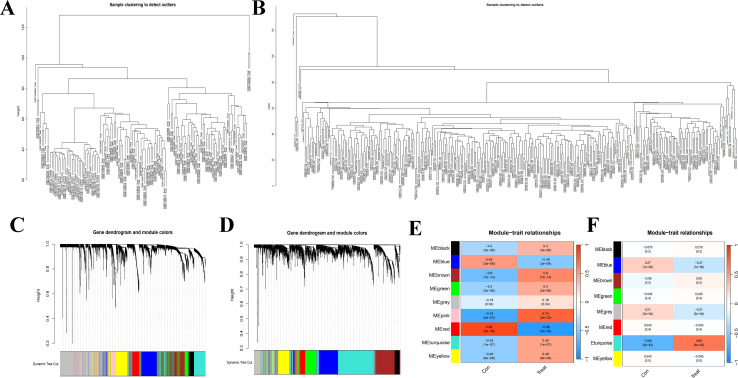
Weighted gene co-expression network analysis (WGCNA). **(A)** Clustering according to the expression level of SLE patients in GSE61635. **(B)** Clustering according to the expression level of PD patients in GSE16134. Each branch represents a sample in the data sets, and there is no outlier sample in each data set. **(C)** Origin and merged modules displaying under the clustering tree for GSE61635. **(D)** Origin and merged modules displaying under the clustering tree for GSE16134. Cluster dendrograms showed the clustering process of the gene modules. **(E)** Heatmap of the correlation between module eigengenes and the occurrence of SLE. **(F)** Heatmap of the correlation between module eigengenes and the occurrence of PD.

**Figure 3:**
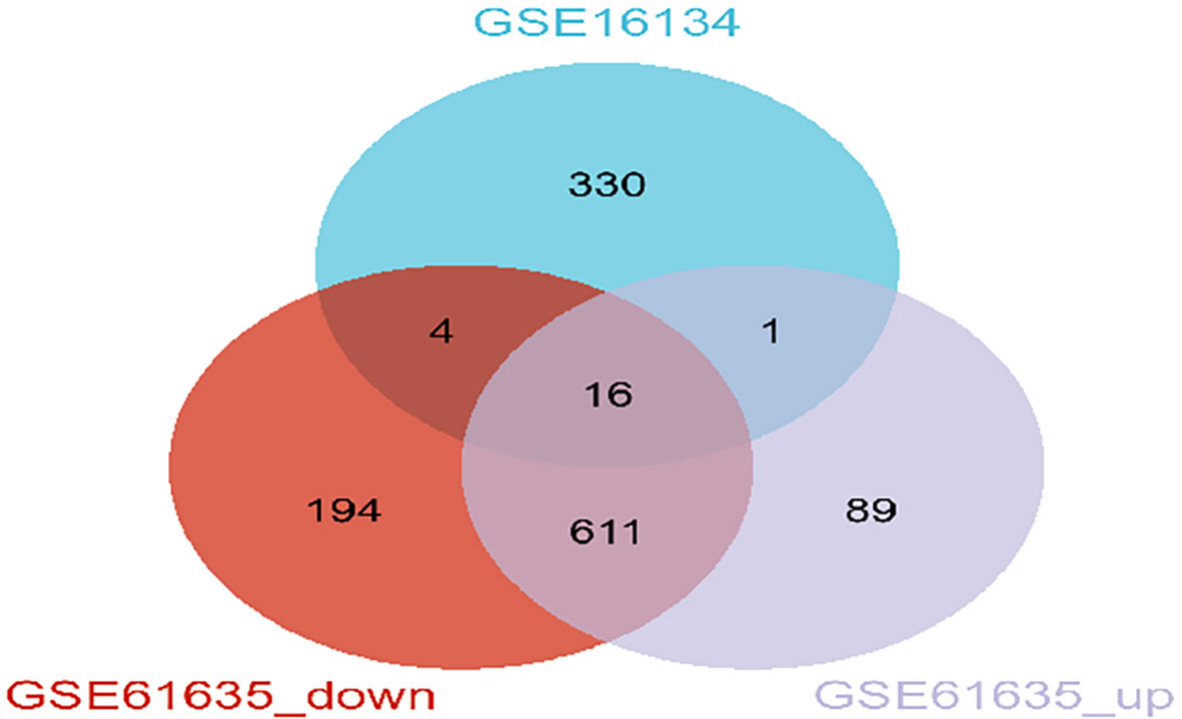
Identification of shared genes between SLE and PD datasets through WGCNA. Veen diagrams indicate that GSE16134 module and GSE61635 module share 21 overlapping DEGs.

The overall study design is shown in **Figure 1**. WGCNA was conducted to investigate the association between clinical traits and genes. All samples from the GSE61635 and GSE16134 datasets were clustered, and no samples were excluded (**Figures 2A, B**). A total of nine modules were identified in the GSE61635 dataset, while eight modules were identified in the GSE16134 dataset. Subsequently, the correlations between the module and clinical traits were computed. The MEpink module had the strongest positive relation with SLE (r = 0.74), followed by brown module (r = 0.6), while the MEred module had the strongest negative relation (r = 0.86) in the GSE61635 database (**Figures 2C, E**). For PD, the MEturquoise module showed the strongest positive correlation (r = 0.68) in the GSE16134 database, while the MEblue module had the strongest negative relation (r = 0.27), followed by grey module (r = 0.21) (**Figures 2D, F**). A total of 21 module genes that overlapped between GSE61635 and GSE16134 were identified (**Figure 3**).

### Enrichment analysis

**Figure 4:**
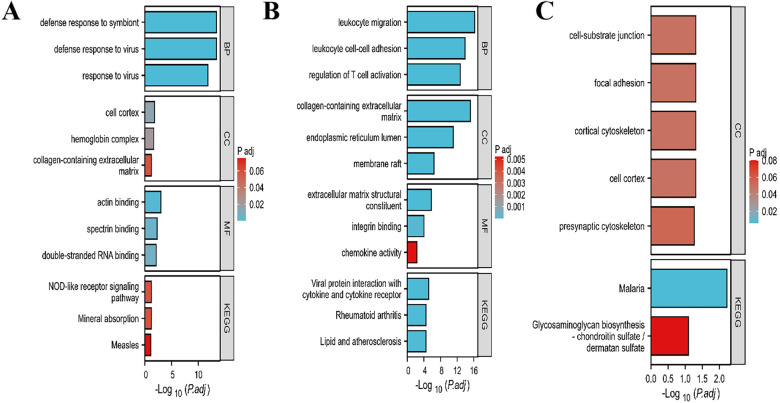
GO and KEGG enrichment analysis of module genes. **(A)** Enriched items of SLE module genes in GO and KEGG analysis. **(B)** Enriched items of PD module genes in GO and KEGG analysis. **(C)** Enriched items of overlapping module genes in GO and KEGG analysis. GO, Gene Ontology; BP, biological process; CC, cellular component; MF, molecular function; KEGG, Kyoto Encyclopedia of Genes and Genomes. p.adjust-value ranking are listed.

For SLE modules, The results of GO/KEGG pathway revealed that module genes were mainly enriched in defense response to symbiont, defense response to virus, response to virus, actin binding, spectrin binding and double-stranded RNA binding (**Figure 4A**). For PD modules, the module genes were mainly enriched in leukocyte migration, leukocyt cell-cell adhesion, regulation of T cell activation, collagen-containing extracellular matrix, endoplasmic reticulum lumen, extracellular matrix structural constituent and viral protein interaction with cytokine and cytokine receptor (**Figure 4B**). For overlapping module genes, we found those feature genes were mainly enriched in malaria and some cell cortex and cell-substrate junction terms (**Figure 4C**).

### Hub gene identification and validation using machine learning algorithms

**Figure 5:**
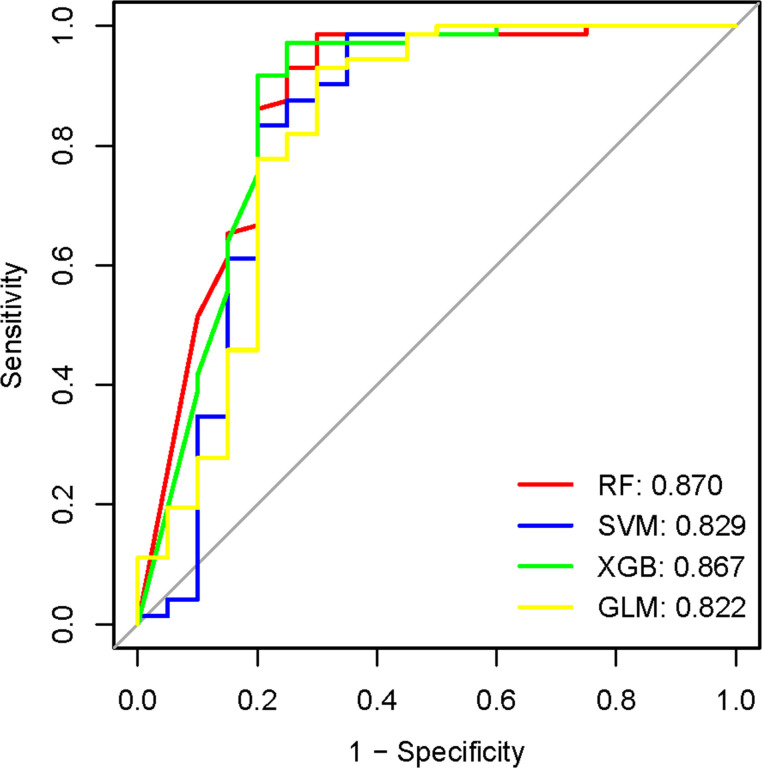
The evaluation of machine learning model performance utilizing the ROC curve. AUC values of the four machine-learning algorithms. RF, Random Forest; SVM, Support Vector Machine; XGB, eXtreme Gradient Boosting; GLM, Generalized Linear Model.

The 21 module genes that overlapped were conducted for difference in the GSE16134 datasets to obtain differentially expressed genes (GSE16134DiffExps), which were then utilized for subsequent analyses. Although each shared hub gene can serve as an auxiliary diagnostic or predictive biomarker, our preference is to develop a comprehensive prognostic model in order to enhance the effectiveness of disease diagnosis and prediction. To further identify the hub genes with the greatest diagnostic value, we selected the most prominent characteristics using machine-learning algorithms. We incorporated the GSE16134DiffExps into four machine learning algorithms (RF, XGB, GLM, and SVM) and obtained the top 10 important genes for each algorithm. The accuracy of the RF algorithm was found to be the highest, as verified by the ROC analysis (**Figure 5**). Consequently, The top 10 genes obtained from the RF algorithm were analyzed for differences in the three datasets (GSE61635, GSE10334, and GSE50772). Ultimately, four hub genes (LY96, EXPH5, RIMS3, and TNEM140) were identified based on their consistent expression patterns across all four datasets.

### PPI network analysis

**Figure 6:**
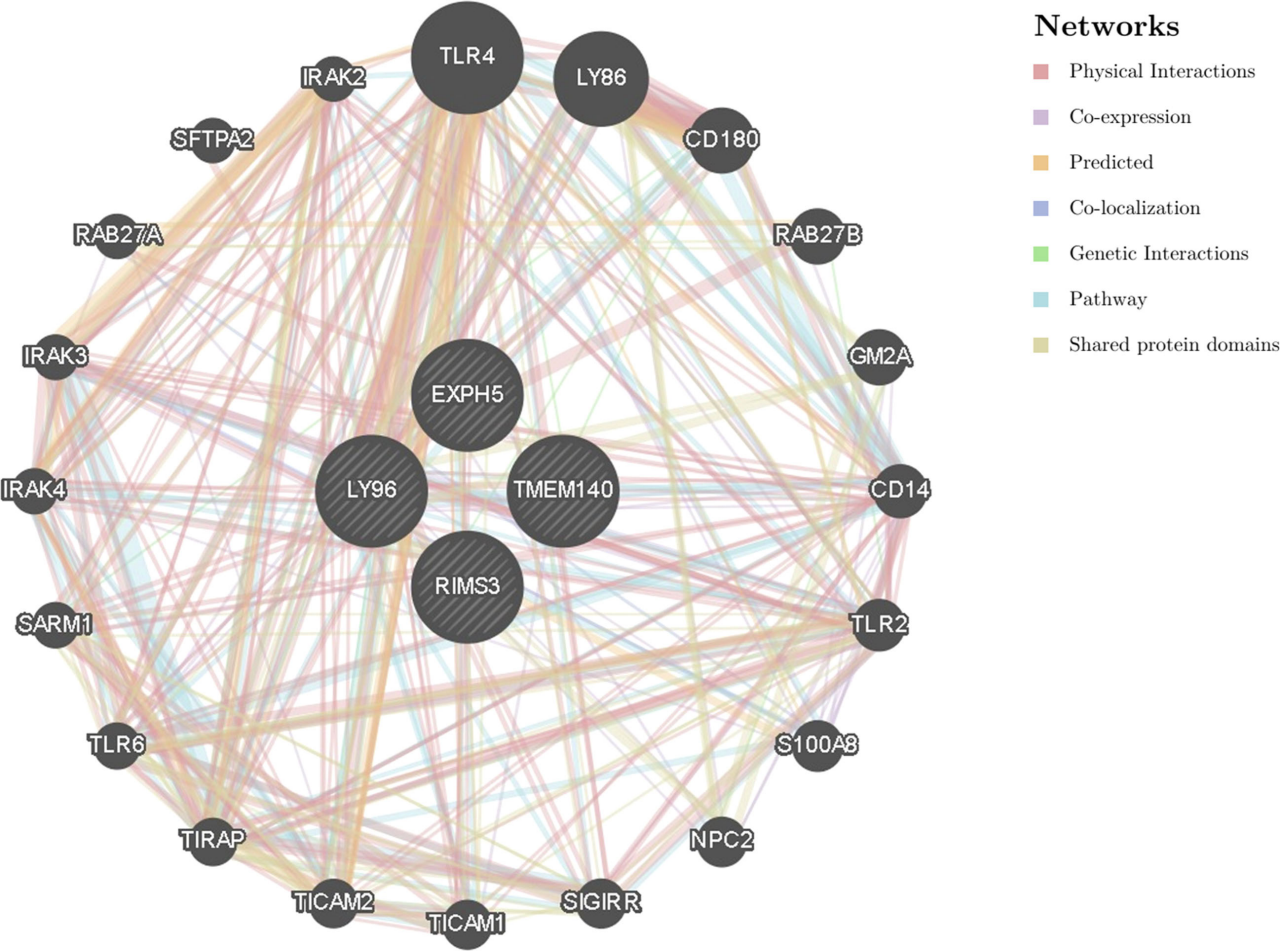
The gene-gene interaction network for hub genes were analyzed using GeneMANIA database. Analysis of hub genes related to the function of LY96, EXPH5, RIMS3 and TMEM140. The 20 most frequently changed neighboring genes are shown. Each node represents a gene. The node color represents the possible functions of the respective gene.

We constructed a gene–gene interaction network for hub genes to analyze the function of these genes using the GeneMANIA database. The hub node representing hub genes were surrounded by 20 nodes representing genes that were significantly correlated with hub genes (**Figure 6**).

### Identification and validation of the hub genes through additional datasets

**Figure 7:**
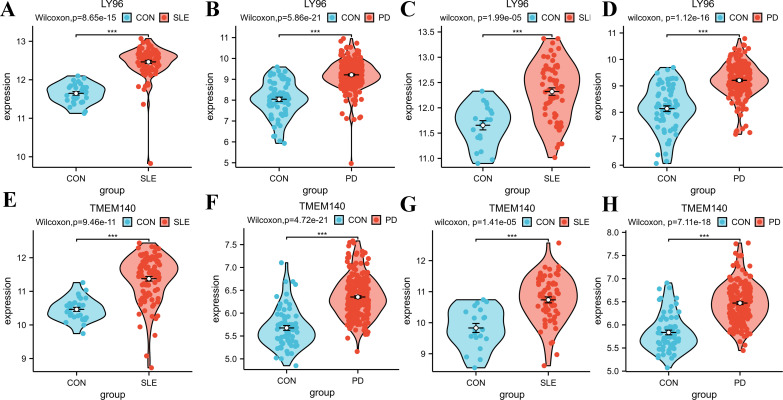
Expression levels of the hub genes in training and Validation datasets. **(A–D)** Violin diagrams of LY96 expression levels in GSE61635, GSE16134, GSE50772, GSE10334; Comparison was conducted by Wilcoxon rank-sum test. **(E–H)** Violin diagrams of TMEM140 expression levels in GSE61635, GSE16134, GSE50772, GSE10334; Comparison was conducted by Wilcoxon rank-sum test. ***p<0.001.

**Figure 8:**
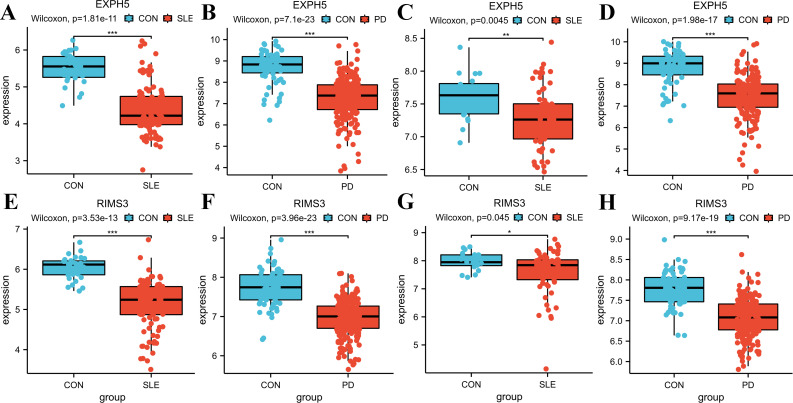
Expression levels of the hub genes in training and Validation datasets. **(A–D)** Boxplots of EXPH5 expression levels in GSE61635, GSE16134, GSE50772, GSE10334; Comparison was conducted by Wilcoxon rank-sum test. **(E–H)** Boxplots of RIMS3 expression levels in GSE61635, GSE16134, GSE50772, GSE10334; Comparison was conducted by Wilcoxon rank-sum test. *p<0.05; **p<0.01; ***p<0.001.

**Figure 9:**
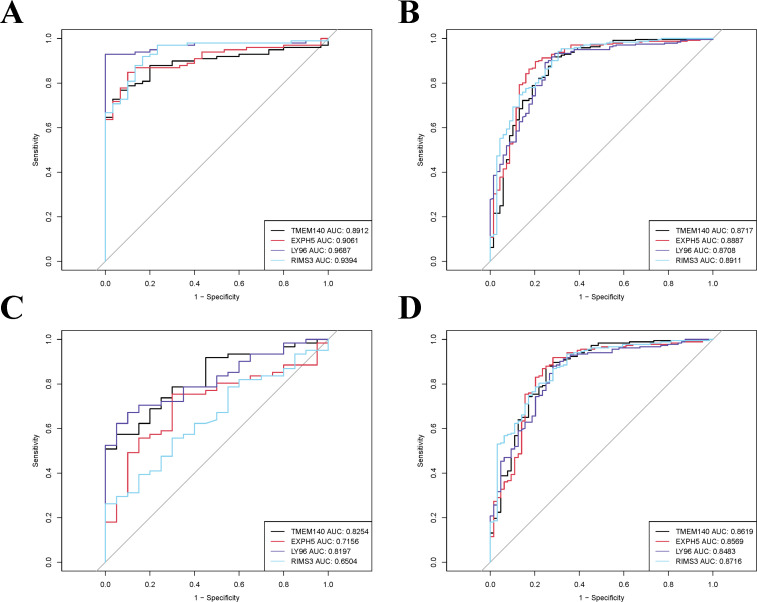
ROC curve analysis of the hub genes in training and validation datasets. **(A, B)** The AUC values of hub genes in training datasets (GSE61635 and GSE16134). **(C, D)** The AUC values of hub genes in validation datasets (GSE50772 and GSE10334).

To validate the accuracy of our findings, we conducted wilcoxon analysis using additional validation datasets for SLE and PD (GSE50772 and GSE10334, respectively). Given that LY96, EXPH5, RIMS3 and TNEM140 have been identified as hub genes, we have chosen these genes for further investigation in order to evaluate their expression from individuals with SLE, PD, and healthy control samples. The expression levels of LY96 and TMEM140 were found to be elevated in the Disease groups, as shown in **Figures 7A–H**. Conversely, **Figures 8A–H** indicate that the expression levels of EXPH5 and RIMS3 were reduced in the Disease groups compared to the control groups. The diagnostic efficacy of each hub gene was further certificated, the 4 genes all exerted a better diagnostic performance through AUC in the four datasets (**Figures 9A–D**).

### Immune cell infiltration and its correlation with hub genes

**Figure 10:**
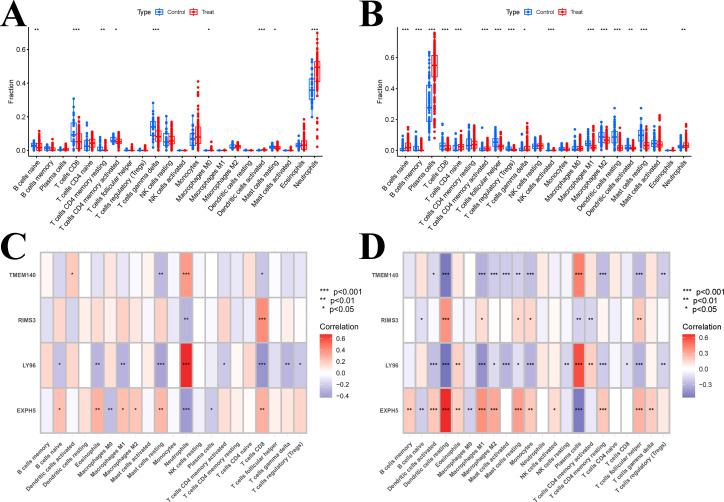
Results of immune infiltration analysis. **(A)** Boxplots of the expression of each immune cell between SLE and control in the GSE61635 dataset. **(B)** Boxplots of the expression of each immune cell between PD and control in the GSE16134 dataset. **(C)** the correlation between hub genes and immune cells by immune infiltration analysis in the GSE61635 dataset. **(D)** the correlation between hub genes and immune cells by immune infiltration analysis in the GSE16134 dataset. Significant difference, *p<0.05, **p<0.01, and ***p <0.001.

We conducted an investigation to determine if it was possible to identify different patterns of immune infiltration using the CIBERSORT method, based on the presence of 22 types of immune cells. Firstly, we conducted an evaluation of the immune cell infiltrate composition in the peripheral blood of two datasets: SLE dataset (GSE61635) and the PD dataset (GSE16134). The boxplot analysis revealed significant differences in the levels of T cells CD8, T cells gamma delta, activated dendritic cells, and neutrophils between the SLE and control samples (**Figure 10A**). CIBERSORT analysis was also conducted on the Periodontal disease data set (GSE16134), The results revealed significant differences between PD and control samples in all cell types, with the exception of T cells CD4 memory resting, NK cells resting, Monocytes, Macrophages M0, Mast cells activated, and Eosinophils (**Figure 10B**). However, the variations in the ratios of immune cell composition are merely one facet of the shared pathogenesis between SLE and PD. We still need to confirm whether these four hub genes are associated with immune infiltration in the peripheral blood. Additionally, it is necessary to determine specifically which immune cells they are associated with and to identify any commonalities among them. Therefore, Pearson correlation analysis was employed to examine the associations between hub genes and immune cells in SLE and PD (**Figures 10C, D**). The results revealed that Neutrophils and Plasma cells exhibited a significant positive correlation with LY96 and TMEM140, while they displayed a negative correlation with EXPH5 and RIMS3 in the GSE61635 and GSE16134 datasets, respectively. Additionally, T cells CD8 and Dendritic cells resting were found to be positively correlated with EXPH5 and RIMS3, but negatively correlated with LY96 and TMEM140 in the GSE61635 and GSE16134 dataset, respectively.

### Metabolic pathway involvement and its correlation with overlapping module genes

**Figure 11:**
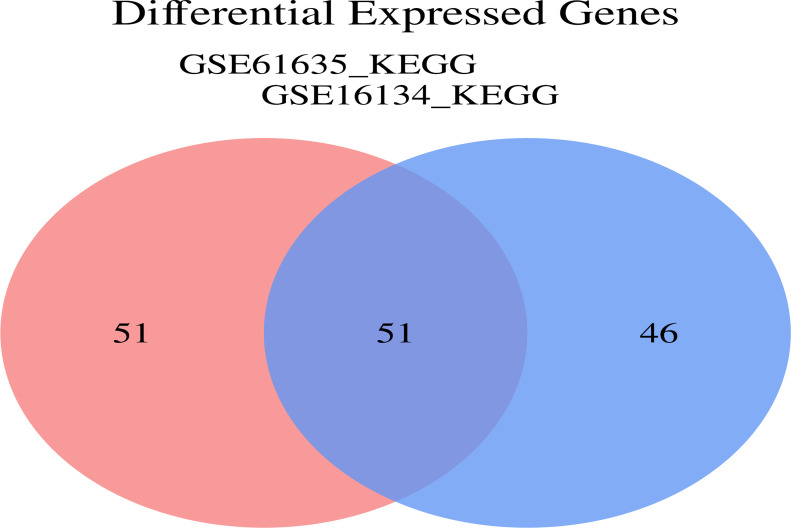
Analysis of GSE61635_KEGG and GSE16134 KEGG. Veen diagrams indicate that GSE16134 KEGG pathway and GSE61635 KEGG pathway share 51 overlapping core pathways.

**Figure 12:**
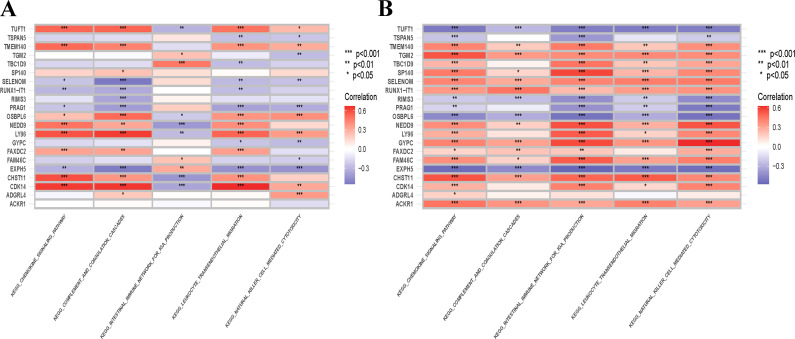
Correlation matrix between metabolic pathways and overlapping module genes in SLE and PD. **(A)** Correlation matrix between metabolic pathways and overlapping module genes in SLE. **(B)** Correlation matrix between metabolic pathways and shared hub genes in PD. The left part showed those overlapping module genes, and the down part showed the metabolic pathways. Red represents for positive correlation, while blue for negative correlation. Asterisks represent levels of signifificance (*p<0.05, **p <0.01, ***p <0.001).

To investigate the functional annotation of the overlapping module genes, the KEGG pathways were obtained via GSVA using microarray expression profiles of the training datasets, and we obtained the cross-pathway using VENNY to obtain the fifty-one core pathways between GSE61635 and GSE16134 in the differential metabolic process (**Figure 11**), From these, the top 5 pathways were selected for further investigation. The GSVA result of the relevant metabolic pathway was visualized in a heatmap. Additionally, a Pearson correlation analysis was conducted to examine the relationship between the overlapping module genes and the top five metabolic pathways. Overall, the findings from the SLE dataset indicate that the chemokine signaling pathway, complement and coagulation cascades and leukocyte transendothelial migration have strong and consistent correlations with overlapping module genes (**Figure 12A**). In the PD dataset, it was observed that the chemokine signaling pathway, intestinal immune network for IGA production and natural killer cell mediated cytotoxicity exhibited strong and consistent correlations with overlapping module genes (**Figure 12B**). Additionally, these pathways exhibited a positive correlation with up-regulated hub genes and a negative correlation with down-regulated hub genes. In other words, the activation of these pathways may occur in SLE and PD.

### Single-cell analysis for the identification of hub genes’ spatial distribution

**Figure 13:**
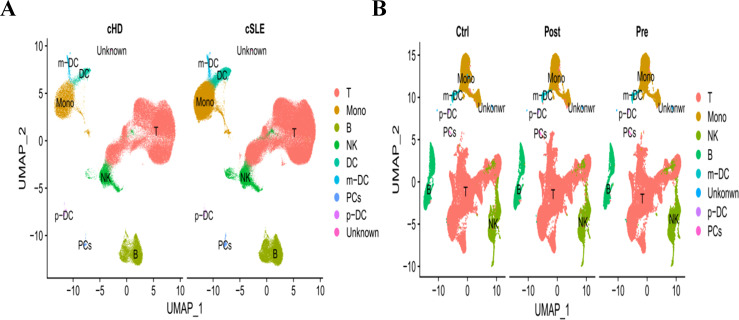
UMAP visualization of SLE and PD single-cell RNA seq datasets. **(A)** UMAP visualization of the 343,618 cells in the single-cell RNA seq dataset GSE135779. **(B)** UMAP visualization of the 115,309 cells in the single-cell RNA seq dataset GSE174609. Different colors indicate distinct clusters; T, B, NK, Mono, m-DC: p-DC and PCs cells.

**Figure 14:**
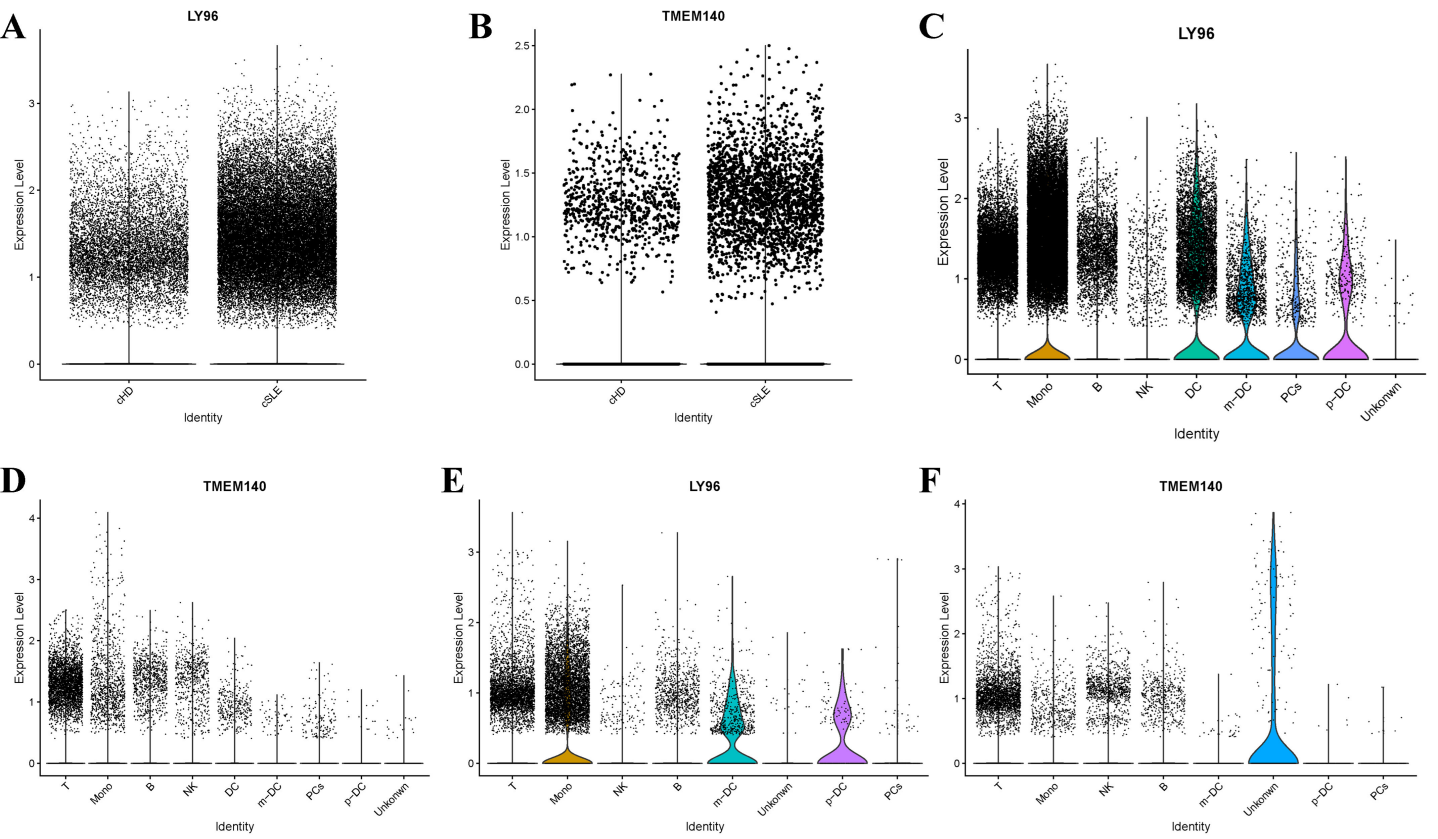
Expression levels of hub genes in GSE135779 dataset and the expression analysis of hub genes in different cell types within GSE135779 and GSE174609. **(A, B)** The expression level of LY96 and TMEM140 in controls and SLE patients in GSE135779. **(C, D)** The expression level of LY96 and TMEM140 in 8 clusters of cells in GSE135779. **(E, F)** The expression level of LY96 and TMEM140 in 8 clusters of cells in GSE174609.

In addition to conducting transcriptomics analysis, we assessed the immune microenvironment of peripheral blood by utilizing scRNA-seq data from GSE135779 and GSE174609. After quality control (QC) procedures, we successfully clustered 343,618 and 115,309 cell populations into a total of 22 distinct clusters, we identified cell populations, Among the identified populations were T cells, monocytes, NK cells, B cells, m-DC, p-DC, PCs and some other cell clusters in the two datasets (**Figures 13A, B**). The findings indicated that there were no neutrophil and plasma cell clusters in samples from individuals with SLE, PD and the control group, This suggests that the subpopulations of these cells may vary. As LY96, TMEM140, RIMS3 and EXPH5 are hub genes, we selected them for further study to assess their expression and localization in PBMC among SLE, PD and normal samples. The expression level of RIMS3 and EXPH5 were both descended in GSE61635 and GSE16134, but no statistical significance in the two scRNA-seq datas. The expression level of LY96 and TMEM140 were both elevated in GSE61635 and GSE16134, which were verified in scRNA-seq datas GSE135779 and GSE174609 (**Figures 14A, B**). Although the two genes are not significantly different in the GSE174609 dataset. In the single-cell datasets of SLE, the analysis revealed that the gene LY96 was predominantly expressed in monocyte, T cell, and dendritic cell clusters, while TMEM140 was primarily expressed in T cell clusters (**Figures 14C, D**). In the single-cell datasets of PD, LY96 was mainly expressed in monocyte and T cell clusters, and TMEM140 was predominantly expressed in T cell clusters (**Figures 14E, F**). This suggests that the differential expression of LY96 and TMEM140 may co-cause SLE and PD through T cell clusters.

### Protein expression of identified hub genes in systemic lupus erythematosus lesions

**Figure 15:**
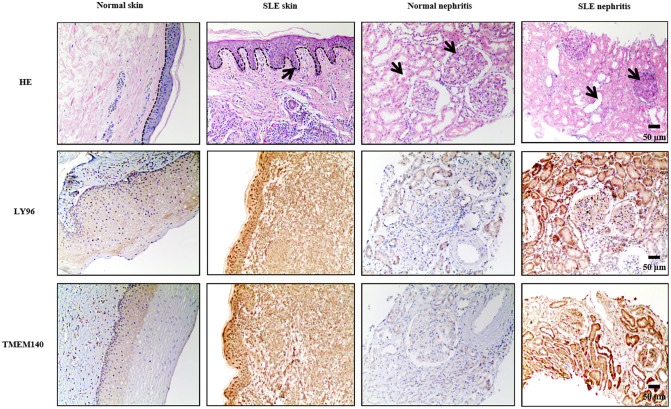
Representative findings on immunohistochemical staining of skin and renal specimens from patients with SLE. LY96 and TMEM140 are upregulated in SLE skin and SLE nephritis tissue. Dotted lines indicate boundaries of dermal-epidermal. The arrows pointed to the regions of pathological alteration. Scale bars = 50 um.

To verify the expression levels of LY96 and TMEM140 in individuals with SLE and healthy controls, we conducted an analysis of their staining using immunohistochemistry. Histological examination through hematoxylin and eosin (HE) staining of the skin and kidneys revealed notable differences between systemic lupus erythematosus (SLE) affected tissues and normal controls. In SLE skin samples, the epidermal papillae exhibited elongation, the basal layer cells of the epidermis demonstrated increased proliferation, and there was a marked increase in inflammatory cell infiltration within the dermis. Similarly, in SLE kidney tissues, the glomerular mesangium showed signs of proliferation and thickening, while the renal tubules were observed to be dilated when compared to control tissues. Immunohistochemical analysis of skin and kidney tissues revealed that, in comparison to the control group, the SLE group exhibited a marked increase in staining intensity. Additionally, there was a greater accumulation of LY96 and TMEM140 in the cased tissues (**Figure 15**). The assessments of histological images clearly demonstrated higher expression levels of LY96 and TMEM140 proteins in tissues from patients with SLE, consistent with the integrative analysis results. This suggests that the positive of LY96 and TMEM140 may be correlated with genes related to diagnosis. Since patients with periodontal disease are rarely examined by pathological sections, we have not verified these two genes in periodontal disease tissues.

### Prediction of potential therapeutic drugs for patients with SLE and PD

**Figure 16:**
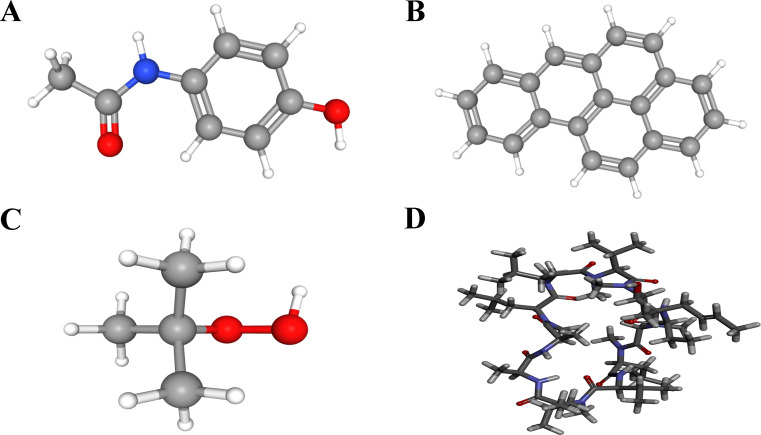
3D structures of small molecule drugs predicted using the PubChem open chemical database, including **(A)** acetaminophen. **(B)** benzo(a)pyreneand. **(C)** tert-Butylhydroperoxide. **(D)** cyclosporine.

Based on the above analysis, LY96 and TMEM140 were possible key genes associated with SLE and PD, Drugs targeting these genes may have a greater impact on the occurrence and progression of the diseases, To confirm their mechanism as molecular drugs for treating patients with SLE and PD, We submitted the two genes(LY96 and TMEN140) to the CTD database to screen for promising small molecule compounds that could be used for SLE and PD management. Using this approach, we identified four potential small-molecule drugs: acetaminophen, benzo(a)pyreneand, tert-butylhydroperoxide and cyclosporine (PubChem number: 1983, 2336, 6410, and 5284373). The structures of these compounds were retrieved from the PubChem database and are displayed in **Figures 16A–D**. Based on the correlation scores between drugs and genes, cyclosporine was selected for subsequent analysis.

### Molecular docking

**Figure 17:**
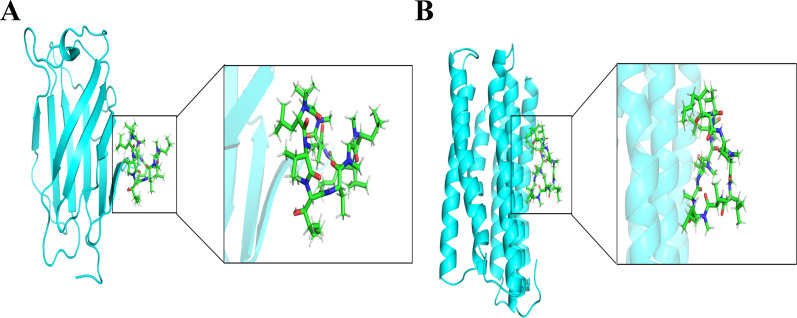
Molecular docking pattern of cyclosporine complexed with the two genes (LY96 and TMEM140). **(A)** LY96. **(B)** TMEM140.

Molecular docking was employed to assess the binding capability of the core targets with their respective compounds. The binding energy between molecules plays a crucial role in determining the effectiveness of molecular docking. A lower binding energy in molecular docking indicates a stronger binding force, a binding energy below -5 kcal/mol suggests favorable binding properties between the receptor and ligand. The docking results (3D structures) are shown in **Figures 17A, B**, and the binding energy is ≤−5 kcal/mol. Based on the aforementioned findings, it can be inferred that the anticipated core targets and their associated active ingredients exhibit a significant or even robust binding capability. the docking score with the genes indicating that the cyclosporine compound had the ability to bind to the active site of genes and potentially exert a positive influence on diseases.

## Discussion

Systemic lupus erythematosus (SLE) is one of the most prevalent systemic autoimmune diseases, which can lead to decreased functional capacity, increased morbidity and mortality. Periodontal disease (PD) is one of the most common oral diseases, characterized by inflamed gums, bleeding and loose teeth. SLE and PD are chronic autoimmune diseases predominantly exhibiting overlapping clinical and serologic characteristics. In a subset of patients with SLE, the disease may advance to exhibit clinical manifestations, serologic profiles, and immunological characteristics that are similar to those observed in PD. As a result, these patients meet the classification criteria for both diseases, giving rise to a condition commonly known as PD/SLE overlap. Despite the presence of clinical evidence indicating potential connections between SLE and PD, the increasing knowledge regarding environmental triggers and epigenetic mechanisms, the genetic factors underlying SLE and PD remain elusive, the precise mechanisms underlying their pathogenesis remain unclear. In this study, we aimed to investigate common target genes, relevant target drugs and action mechanism in SLE and PD through integrative bioinformatic analyses of transcriptomes.

We performed integrative bioinformatics analysis in combination with machine learning algorithms to identify hub genes, pathways and moluler in SLE and PD. Firstly, we conducted an analysis of coexpression modules in SLE and PD. We then identified the intersection of the relevant modules, resulting in the discovery of 21 shared candidate genes. Finally, 4 hub genes (LY96, TMEM140, EXPH5 and RIMS3) were screened, which have consistent expression trends in training and validation datasets.

Inflammation can stimulate leukocyte transendothelial migration via their display of cytokines, chemokines, complement and angiogenesis factors, and Cell adhesion, migration, and immunity are key steps for pathogenicity in disease progression (27–31). The immune responses not only Involved in the pathological mechanism of SLE but also affect bone remodeling through impact on osteoblastlineage cells, periodontal ligament, fibroblasts and osteoclasts, which impact bone resorption and bone coupling in PD. In SLE, complement can serve as an important source of molecules to signal ‘danger’ and form pathogenic immune complexes (32). In PD, Osteoclast precursors are recruited to sites of inflammation by chemokines, Some chemokines support the proliferation of precursors or their differentiation to osteoclasts (33). Osteoclasts also produce chemokines to amplify osteoclastogenesis and bone resorption (34). Consistent with GSVA of the our study, the Metabolic pathway are mainly Immune-related and inflammatory-related, we found that these data provided useful information explaining how shared genes implicated in disease progression, especially the 4 hub genes, although more in-depth studies are necessary.

CIBERSORT is an inverse convolution analysis algorithm based on linear support vector regression that estimates the relative abundance of immune cells in a mixed cell population by analyzing gene expression data (35). Using this algorithm, we found that there was increased infiltration of Neutrophils, Dendritic cells activated in SLE samples, and decreased infiltration of T cells CD8 and T cells gamma delta, there was increased infiltration of T cells CD4 memory activated, there was increased infiltration of Plasma cells, T cells CD4 naive and B cells naive in PD samples, and decreased infiltration of B cells memory, T cells CD8, T cells follicular helper, T cells regulatory, NK cells activated, Dendritic cells resting, Macrophages M1, Macrophages M2 and Mast cells resting. Many studies have indicated that SLE may lead to a higher risk of periodontitis, as SLE is characterized by immune system dysregulation, with overactive phagocyte cells and elevated production of pro-inflammatory cytokines, such as interleukin (IL)-1b and IL-18, which may have pathogenetic roles in periodontitis (36–38). Moreover, oral infection is also a common side effect of the use of cortisol and immunosuppressants in the treatment of SLE (39). In contrast, periodontitis could also promote the occurrence and development of SLE, and several theories have been proposed as potential explanations for this association, Like our immune infiltration results, in patients with periodontitis, plasma cells are increased and represent almost half of infiltrated immune cells in periodontal lesions, which are considered to play a crucial role in SLE pathogenesis (40). Thus, we speculate that immune environment generates an inflammatory environment, Released pro-inflammatory cytokines promote the infiltration of some immune cells, regulate hub gene expression, maintain the inflammatory microenvironment, and ultimately exacerbate SLE and PD.

Since there was no significant difference expression in RIMS3 and EXPH5, we only studied LY96 and TMEM140 further, in our study, LY96 mainly distributed in monocytes, DC and T lymphocytes and TMEM140 was mainly expressed in T lymphocytes cluster in SLE samples, and The expression level of LY96 and TMEM140 were both elevated Compared with control group, which is consistent with our research above. We did the same analysis on periodontal samples, LY96 was highly expressed in monocytes and T lymphocytes clusters, TMEM140 was mainly expressed in T lymphocytes clusters, Although the expression levels of the two genes were not significantly different from those of the control group. Accumulating evidence suggests that T cells and cytokines play a crucial role in the pathogenesis of SLE, The balance among T cell subpopulations is of utmost importance, An imbalance of T cell subsets has been implicated in the development of SLE (41, 42). in addition, T cells are central in regulating immune-mediated mechanisms and in the pathogenesis of PD. Our single-cell sequencing findings revealed an increased abundance of T lymphocytes. Consequently, we hypothesized that LY96, TMEM140 and T lymphocytes may serve as the shared pivotal genes and immune cells in both SLE and PD.

## Conclusion

We identified central genes through multiple bioinformatics methods and established effective diagnostic models for SLE and PD. By comparing with external gene sets, four genes, LY96, TMEM140, RIMS3 and EXPH5, were identified as hub genes. Immunohistochemical (IHC) techniques and single-cell sequencing further confirmed the increased protein expression levels of LY96 and TMEM140 in human SLE lesions compared with normal control tissues. We further analyzed the drugs that are sensitive for the treatment of these two genes. Our study shows that LY96 and TMEM140 genes are expected to be shared key biomarkers for the diagnosis and treatment of SLE and PD, which not only provides clinical basis for the diagnosis and treatment of single systemic lupus erythematosus or periodontal disease, but also an ideal diagnosis and treatment indicator for overlapping diseases of the two.

## Data Availability

The original contributions presented in the study are included in the article/supplementary material. Further inquiries can be directed to the corresponding authors.
